# Evaluation of a Six Sigma‐Based Dynamic Quality Control Strategy for Hematology Analysis: A Multicenter Study

**DOI:** 10.1002/jcla.70138

**Published:** 2025-12-21

**Authors:** Bo Liu, Zhaodong Sun, Kaiyong Chen, Na Wang, Jibao Qin, Dengli Feng, Fumeng Yang, Jiaping Wang, Huiyi Wu, Ming Hu

**Affiliations:** ^1^ The First Affiliated Hospital of Kangda College of Nanjing Medical University Lianyungang Jiangsu China; ^2^ Department of Laboratory Medicine Guanyun Hospital Affiliated to Kangda College of Nanjing Medical University Lianyungang Jiangsu China; ^3^ Department of Laboratory Medicine Donghai Hospital Affiliated to Kangda College of Nanjing Medical University Lianyungang Jiangsu China; ^4^ Department of Laboratory Medicine Lianyungang Oriental Hospital Lianyungang Jiangsu China; ^5^ Department of Laboratory Medicine Lianyungang Maternal and Child Health Hospital Lianyungang Jiangsu China; ^6^ Department of Laboratory Medicine Lianyungang Second People's Hospital Lianyungang Jiangsu China

**Keywords:** dynamic quality control, hematology testing, LSTM, moving average, six sigma

## Abstract

**Background:**

Quality control (QC) is critical for ensuring the accuracy and reliability of hematology testing. Traditional QC strategies, however, are often limited in their ability to provide timely detection of analytical errors and to adapt to complex, real‐world laboratory conditions.

**Methods:**

In this multicenter study, we applied the Six Sigma quality management framework to systematically evaluate the performance of five hematology parameters (Hb, WBC, RBC, HCT, and PLT). To enhance QC monitoring, we established a dynamic quality control strategy that integrates moving average (MA) monitoring with a long short‐term memory (LSTM) predictive model. Patient sample data were incorporated alongside routine QC data to validate clinical adaptability.

**Results:**

Sigma metrics revealed marked performance differences among the parameters, with Hb and WBC achieving world‐class or excellent performance (*σ* ≥ 6), while PLT showed relatively lower stability. The combined MA–LSTM approach significantly improved sensitivity for error detection while reducing false positives compared with conventional rule‐based QC. The dynamic model demonstrated robust predictive ability, enabling real‐time QC monitoring across multiple laboratory sites.

**Conclusion:**

By combining Six Sigma evaluation, MA monitoring, and LSTM modeling, we propose a dynamic QC strategy that overcomes key limitations of conventional quality control methods. This approach provides laboratories with an intelligent, proactive, and clinically adaptable solution for improving the reliability of hematology testing and ensuring higher quality patient care.

## Graphical abstract

This study employed the Six Sigma quality management framework to evaluate the performance of hematology parameters across multiple centers and established a dynamic quality control strategy by integrating moving average (MA) and long short‐term memory (LSTM) methods. The proposed approach enhanced error detection sensitivity, reduced false positives, and enabled more intelligent, real‐time laboratory quality management.

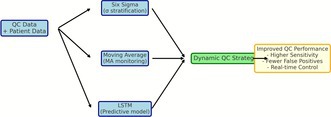

## 1 Introduction

Hematology analysis is among the most frequently performed tests in clinical laboratories and is indispensable for diagnosing and monitoring a wide range of pathological conditions, including infections, inflammation, anemia, bleeding disorders, and hematologic diseases. With advances in automation, modern hematology analyzers now offer high‐throughput, multiparameter, and multimodal testing, substantially improving both efficiency and standardization [1, 2]. However, in daily quality management, different hematology parameters vary in allowable total error (TEa), analytical stability (CV), and clinical risk level, yet existing quality control (QC) strategies often fail to account for these differences. Most laboratories continue to apply uniform QC frequencies and rules in a “one‐size‐fits‐all” approach, which can waste resources on high‐performance parameters and delay error detection for low‐performance ones, ultimately compromising both safety and timeliness [3, 4]. This problem is particularly acute in primary and regional laboratories, where limited manpower and resources frequently result in redundant QC for robust assays and insufficient monitoring of weaker ones [5, 6, 7].

In recent years, the Six Sigma quality management framework has been widely adopted in clinical laboratories to achieve more precise and quantitative performance evaluation [8, 9, 10, 11]. International guidelines, including those from ISO and CLSI, explicitly recommend risk‐based, differentiated QC strategies tailored to analytical performance (e.g., sigma values) to improve efficiency and optimize resource allocation [12, 13]. Previous studies have shown that sigma‐guided dynamic QC models can enhance sensitivity while reducing unnecessary workload [14, 15]. Westgard and colleagues further proposed stratified QC strategies: assays with high performance (*σ* ≥ 6) could be monitored with reduced frequency and simplified rules, whereas assays with poor performance (*σ* < 3) required increased QC frequency and multirule approaches to strengthen error detection [16]. In routine assays such as glucose and electrolytes, dynamic QC has been demonstrated to significantly improve bias detection rates and shorten response times, sometimes enabling recognition of out‐of‐control events 1–3 h earlier than conventional strategies [4, 17].

Despite this progress, systematic research on Six Sigma‐based dynamic QC strategies in hematology remains lacking. Hematology parameters such as WBC, Hb, and PLT differ markedly in clinical importance, analytical stability, and permissible error, leading to wide variation in sigma levels. This underscores the need to establish individualized QC pathways for hematology testing.

The rapid development of artificial intelligence (AI) has created new opportunities for QC innovation. The moving average (MA) algorithm, which generates real‐time trend curves from patient data, helps overcome the limitations of traditional QC materials with low testing frequency and delayed responses. It has already been validated in assays such as glucose and electrolytes [4, 17, 18]. Similarly, long short‐term memory (LSTM) networks, a type of deep learning model, excel at capturing long‐term dependencies in time‐series data and have been applied in laboratory data modeling and anomaly detection [19]. Studies suggest that LSTM can provide early warnings before biases exceed QC limits, greatly enhancing QC sensitivity and predictive capability [20, 21]. However, applications of MA and LSTM in hematology QC are still in their infancy, and supporting evidence is limited.

Globally, laboratory QC is moving from fixed protocols toward risk‐based and differentiated management, as reflected in initiatives such as IQCP by the College of American Pathologists [22] and similar efforts in the United Kingdom [23]. However, systematic applications of such strategies in hematology remain limited. To address this gap, we designed the present study with three hypotheses: (1) hematology parameters show wide variation in sigma levels, necessitating differentiated QC strategies [15, 16]; (2) sigma‐based dynamic QC can improve efficiency without increasing resource consumption [14, 17]; and (3) the integration of moving average (MA) algorithms [17, 18] and long short‐term memory (LSTM) models [19, 20, 21] enables earlier and more accurate detection of systematic bias. To our knowledge, this represents the first application of MA and LSTM in hematology QC, extending beyond their prior use in biochemical assays.

Based on these considerations, the present study was designed with the following hypotheses: (1) hematology parameters show significant variation in sigma levels, necessitating differentiated QC strategies; (2) sigma‐based dynamic QC can improve efficiency without increasing resource consumption; and (3) the incorporation of MA and LSTM models allows earlier and more accurate detection of systematic bias compared with conventional Westgard rules. By validating these hypotheses in a multicenter study, we aim to provide evidence to support the development of individualized and intelligent QC pathways for hematology testing. To our knowledge, this is the first study to integrate MA algorithms with LSTM predictive modeling for hematology QC, representing a departure from prior applications confined to biochemical assays.

## 2 Materials and Methods

### 2.1 Study Design and Participants

This prospective multicenter study was conducted in six clinical laboratories across the Lianyungang region. All sites were equipped with Mindray BC‐7500 series hematology analyzers and followed a standardized QC protocol. The study was carried out from August 2023 to December 2024. All QC data were anonymized prior to analysis and derived from retrospective QC records. Given the nature of the data, the institutional review boards of the participating centers granted an exemption from formal ethics approval [24].

### 2.2 Analytical Parameters and QC Procedures

The study focused on five routine hematology parameters: white blood cell count (WBC), red blood cell count (RBC), hemoglobin (Hb), hematocrit (HCT), and platelet count (PLT). All centers used the same lot of internal QC materials, and data were collected twice daily over a 6‐month period. The total allowable error (TEa) limits for each parameter were defined according to the Clinical Laboratory Improvement Amendments of 1988 (CLIA ‘88) [24].

### 2.3 Calculation of Bias, Imprecision, and Sigma Metrics

Analytical performance was assessed using the Six Sigma quality management framework [15]. Sigma metrics (*σ*) were calculated as:

*σ* = (TEa—Bias) / CV, where: TEa (Total Allowable Error): the maximum permissible error defined by CLIA ‘88 [24]; Bias: the systematic deviation between the laboratory mean and the assigned reference value; CV (Coefficient of Variation): the measure of imprecision, calculated as CV = (SD/Mean) × 100%, with SD representing the standard deviation during the 6‐month stability period and Mean, the corresponding mean value.

Bias was expressed as a percentage: Bias (%) = |(Laboratory Mean—Reference Value)/Reference Value| × 100%.

To ensure standardization and traceability, the reference value for all parameters was defined as the all‐laboratory median from the National Center for Clinical Laboratories (NCCL) external quality assessment (EQA) program [24], specifically from peer laboratories using the Mindray BC‐7500 platform. This approach, recommended by CLIA and consistent with ISO 15189:2022 [24], provides both representativeness and metrological traceability. For parameters with lower sigma values (e.g., PLT), additional validation was performed using allowable biological variation data from the Biological Variation Database (https://biologicalvariation.eu) [24] to confirm the appropriateness of reference values.

Sigma values were categorized as follows [15]: *σ* ≥ 6: world‐class quality; 3 ≤ *σ* < 6: acceptable quality; *σ* < 3: high‐risk performance, requiring more frequent QC and multirule monitoring.

Cross‐center harmonization: Several measures were adopted to ensure comparability of sigma metrics across centers: (1). All laboratories used Mindray BC‐7500 analyzers under stable operating conditions. Calibration was performed according to the manufacturer's recommendations to minimize variability [24], minimizing platform variability. (2). Identical lots of reagents and QC materials were distributed to all sites, eliminating lot‐to‐lot variation. (3). Bias estimation was standardized using the NCCL EQA all‐laboratory median from the Mindray peer group [24], ensuring representativeness and traceability.

4. Before formal data collection, two rounds of inter‐laboratory comparison were conducted. All parameter‐specific bias differences were < 3%, fulfilling the prerequisite for reliable cross‐center sigma evaluation.

#### 2.3.1 Supplemental validation using patient data

To enhance the clinical relevance of sigma (*σ*) evaluation and to verify the consistency of performance between QC materials and patient samples, test data were randomly extracted from the laboratory information systems (LIS) of six participating centers. A continuous 30‐day dataset was selected, covering five hematology parameters: WBC, RBC, Hb, HCT, and PLT. Before inclusion, the following sources of interference were excluded: (1) extreme values exceeding the medical decision limit (MDL) by ±3 SD; and (2) samples flagged for hemolysis, lipemia, or clotting, indicative of pre‐analytical errors.

The coefficient of variation for patient samples (CV_patient) was calculated as:

CV_patient = (SD_patient/Mean_patient) × 100%. To account for potential matrix effects between QC materials and clinical specimens, a matrix correction factor (F) was introduced:

$$\begin{array}{c} F=\mathrm{CV}\_\text{patient}/\mathrm{CV}\_\mathrm{IQC}.\\ \;\text{The adjusted sigma value was then calculated}\;\mathrm{as}:\\ \sigma \_\text{adjusted}=\left(\mathrm{TEa}-\text{Bias}\right)/\left(\mathrm{CV}\_\mathrm{IQC}\times F\right) \end{array}$$

This method compensates for potential discrepancies between QC materials and clinical specimens, thereby improving the representativeness and applicability of sigma metrics in real‐world practice. It is particularly useful for parameters with high biological variability, such as platelet count (PLT).

### 2.4 Design of the Dynamic QC Strategy

Based on sigma stratification, a differentiated dynamic QC strategy was developed:

*σ* ≥ 6 (high‐performance parameters): QC performed once daily, applying a single 1–2 s rule.

3 ≤ *σ* < 6 (moderate‐performance parameters): QC performed for each analytical batch, using multirule combinations (e.g., 1–2 s, R–4 s).

*σ* < 3 (low‐performance parameters): QC performed at an increased frequency (every 4 h), with dual‐rule monitoring.

**FIGURE 1:**
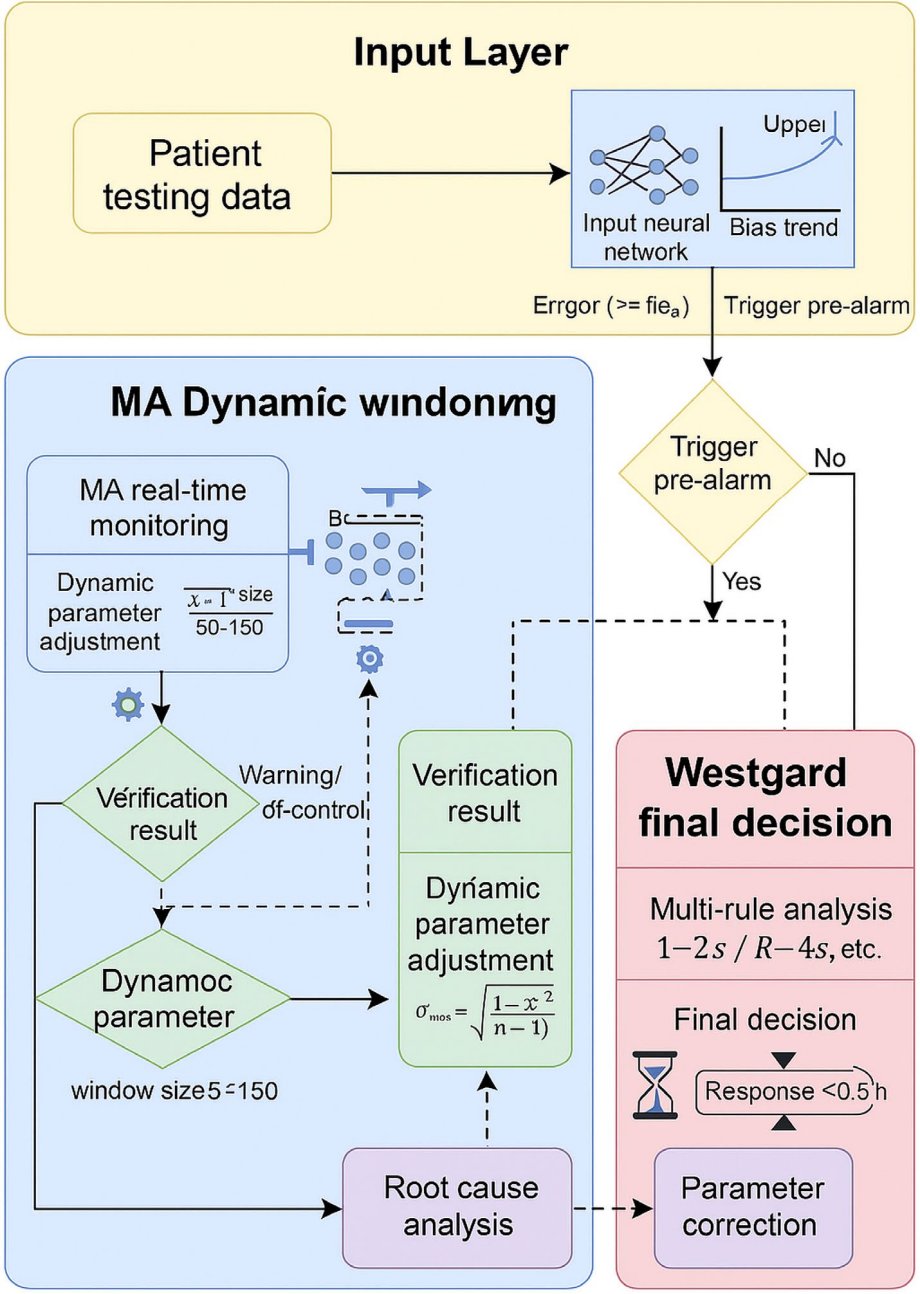
Collaborative logic between MA and Westgard rules.

In addition, several centers implemented the moving average (MA) algorithm as a supplementary monitoring tool. The MA algorithm provided continuous surveillance of patient data and offered prospective alerts, while conventional Westgard rules served as a secondary verification step. Together, these measures established a dual safeguard mechanism of “screening–verification” (Figure 1).

### 2.5 Design of MA‐Based Monitoring Logic and Dynamic Window Adjustment

To meet the practical requirements of *σ*‐stratified quality management in hematology analysis, particularly for high‐risk parameters with *σ* < 3 (e.g., platelet count, PLT), we established a moving average (MA)‐based monitoring framework. This system integrates a dynamic sliding window strategy, aiming to enhance real‐time sensitivity while maintaining stability.

Parameter settings for MA were determined according to both published recommendations and pilot experiments. The baseline window size was defined as 100 consecutive patient samples, in line with the guidelines proposed by Sciacovelli et al. [25, 26]. Comparative analyses of 50‐, 100‐, and 150‐sample windows demonstrated that the 100‐sample configuration achieved the most favorable balance, with sensitivity ≥ 80% and a false‐positive rate < 15%.

To accommodate temporal variability in patient data, we further designed an adaptive window adjustment mechanism:

When the real‐time coefficient of variation (CV) exceeded the historical CV, the window size was reduced to 50 samples to strengthen responsiveness to short‐term shifts.

Conversely, when the real‐time CV was less than or equal to the historical CV, the window was expanded to 150 samples to mitigate spurious alarms caused by isolated outliers.

Dynamic control limits were defined as target mean ± (1.0–1.8) × SD. Following validation, ±1.5 SD was adopted as the final alert threshold, which yielded the optimal trade‐off between sensitivity and specificity.

#### 2.5.1 Sliding Window and CV‐Driven Adjustment Mechanism

Baseline window: 100 consecutive patient samples; Dynamic adjustment: reduced to 50 samples if real‐time CV > historical CV, expanded to 150 samples if real‐time CV ≤ historical CV; Control limits: alert limit = target mean ± (1.0–1.8) × SD; out‐of‐control limit = target mean ± 2.5 SD. These thresholds were integrated with Westgard multirules to establish a dual‐layer verification mechanism.

#### 2.5.2 Implementation Protocol for High‐Risk Parameters (PLT)

For parameters with *σ* < 3, such as PLT, certain centers (A, C, and D) incorporated MA monitoring as a pre‐screening tool prior to the application of Westgard rules. The protocol included the following elements:

Data preprocessing: exclusion of extreme values exceeding ±3 SD to ensure a robust mean estimate.

Linked rules: MA‐triggered alerts were subsequently verified using Westgard multirules (e.g., 1–2 s or R‐4 s).

Corrective actions: if the MA exceeded the alert threshold for three consecutive runs, or exceeded the out‐of‐control limit on a single occasion, testing was suspended immediately, followed by a structured root‐cause investigation (e.g., reagent lot verification, instrument recalibration).

### 2.6 LSTM‐Based Predictive Framework and Early‐Warning Trigger Mechanism

To enable proactive detection of systematic shifts, a long short‐term memory (LSTM) network was integrated upstream of the MA algorithm, thereby establishing a three‐tier collaborative framework of trend prediction–dynamic monitoring–rule verification.

The LSTM model was configured as follows:

Input dimension: 100 consecutive PLT test values, ensuring adequate data for trend learning.

Hidden layer architecture: 64 neurons; selected after comparing 32, 64, and 128 units to balance predictive accuracy and overfitting risk.

Activation function: *tanh*, which demonstrated superior convergence compared with *sigmoid* and *ReLU* on the present dataset.

Data partitioning: 80% of samples were used for training and 20% for validation to enhance generalizability.

Prediction horizon: 10 subsequent samples; this setting provided an optimal compromise between predictive accuracy and lead time, achieving an average lead time of ~3 h compared with 5‐ or 20‐sample forecasts.

Early‐warning threshold: triggered when the predicted deviation exceeded 50% of the allowable total error (TEa). Pilot testing confirmed that this threshold achieved an AUC ≥ 0.85, indicating satisfactory accuracy and timeliness.

#### 2.6.1 LSTM Model Architecture

Input layer: receives 100 consecutive PLT measurements.

Hidden layer: 64 neurons with *tanh* activation.

Output layer: predicts the mean deviation trend of the next 10 samples.

Training dataset: 6‐month historical QC database; training/validation split of 8:2.

Loss function: mean absolute error (MAE).

The model was trained using an 80:20 split with five‐fold cross‐validation. Early stopping was applied to prevent overfitting.

#### 2.6.2 Early‐Warning Trigger Mechanism

When the LSTM model predicts that the deviation trend of consecutive samples will exceed 50% of TEa, the system immediately issues a red alert, which is automatically relayed to quality control personnel. This allows early initiation of corrective interventions before conventional QC rules are breached.

### 2.7 Evaluation of Intervention Effectiveness

The effectiveness of the dynamic QC strategy was assessed by comparing pre‐ and post‐implementation outcomes across the following indicators:

Bias detection rate.

Response time to out‐of‐control events.

Frequency of QC testing and associated costs.

To evaluate inter‐center consistency, Kappa statistics were applied.

Categorization of outcomes: changes were classified as “improved” or “no improvement/decline.”

Criteria for improvement: defined as ≥ 2% increase in bias detection rate, ≥ 1 h reduction in response time, or ≥ 10% increase in intervention rate.

Consistency analysis: 2 × 2 contingency tables were constructed to calculate κ values. A κ > 0.75 was considered strong agreement, 0.40–0.75 moderate agreement, and < 0.40 poor agreement.

Statistical analysis: performed with the Crosstabs module in SPSS 26.0, with 95% CI estimation; *p* < 0.05 was regarded as statistically significant.

Definitions of QC Events.

Alerting Event: defined as a shift detected by the MA or LSTM model that exceeds predefined thresholds (e.g., target ±1.5 SD for MA, or LSTM‐predicted deviation > 50% TEa) but not yet confirmed by conventional Westgard rules. These events emphasize trend recognition and proactive intervention.

Out‐of‐Control Event: defined as fulfillment of any Westgard failure rule (e.g., 1–2 s, R–4 s) requiring actual QC intervention (e.g., recalibration, test suspension, and reagent replacement). Alerts generated by MA or LSTM that are subsequently confirmed by Westgard rules were also classified as out‐of‐control events.

Furthermore, for combined trigger mechanisms (MA + Westgard), alerting events that ultimately satisfied Westgard criteria were simultaneously recorded as out‐of‐control events. This dual classification allowed evaluation of the positive predictive value of MA‐based early alerts.

### 2.8 Statistical Analysis

All statistical analyses were performed using SPSS version 26.0 (IBM Corp., Armonk, NY, USA). Data with a normal distribution are expressed as mean ± standard deviation and compared using the paired t‐test. Non‐normally distributed data are presented as median (interquartile range) and analyzed with the Wilcoxon signed‐rank test. Differences in *σ* values among multiple centers were assessed using one‐way analysis of variance (ANOVA) or the Kruskal–Wallis H test, as appropriate. When overall differences were statistically significant, pairwise comparisons with Bonferroni correction were performed. Inter‐center agreement was evaluated using the Kappa statistic. All tests were two‐sided, and a *p* < 0.05 was considered statistically significant.

## 3 Results

### 3.1 Evaluation of *σ* Levels

Six tertiary hospitals (designated A–F) were included in this study. All centers employed Mindray BC‐7500 hematology analyzers with automated QC functions and utilized the same lot of control materials, ensuring cross‐center comparability and consistency. Sigma (*σ*) values were calculated and evaluated for five hematological parameters (WBC, RBC, Hb, HCT, and PLT). The results are summarized as follows:

Hemoglobin (Hb): All six centers achieved *σ* > 6, with a mean of 6.87 (range: 6.31–7.42), meeting the “world‐class” quality benchmark. This indicates high system stability with minimal bias and variability.

White blood cell count (WBC): *σ* values ranged from 6.10 to 7.05 (mean 6.59). Except for Center F (5.98), all centers maintained *σ* ≥ 6, suggesting consistently high analytical performance.

Red blood cell count (RBC): *σ* values varied between 4.28 and 5.73 (mean 4.98), representing an acceptable level of performance. However, the relatively large inter‐center CV differences highlight the need for enhanced instrument maintenance and cross‐site harmonization.

Hematocrit (HCT): *σ* values ranged from 4.11 to 5.89 (mean 4.65). Bias levels were generally between 1.2% and 1.9%, indicating slightly lower stability compared with RBC, though still within acceptable quality limits.

Platelet count (PLT): PLT exhibited the lowest *σ* values among the five parameters, ranging from 2.58 to 3.74 (mean 3.19). Only Centers A and B reached *σ* ≥ 3.5, whereas the remaining centers demonstrated either excessive bias or elevated CV. These findings identify PLT as the highest‐risk parameter, warranting priority monitoring, increased QC frequency, and dynamic management incorporating multirule approaches.

**FIGURE 2:**
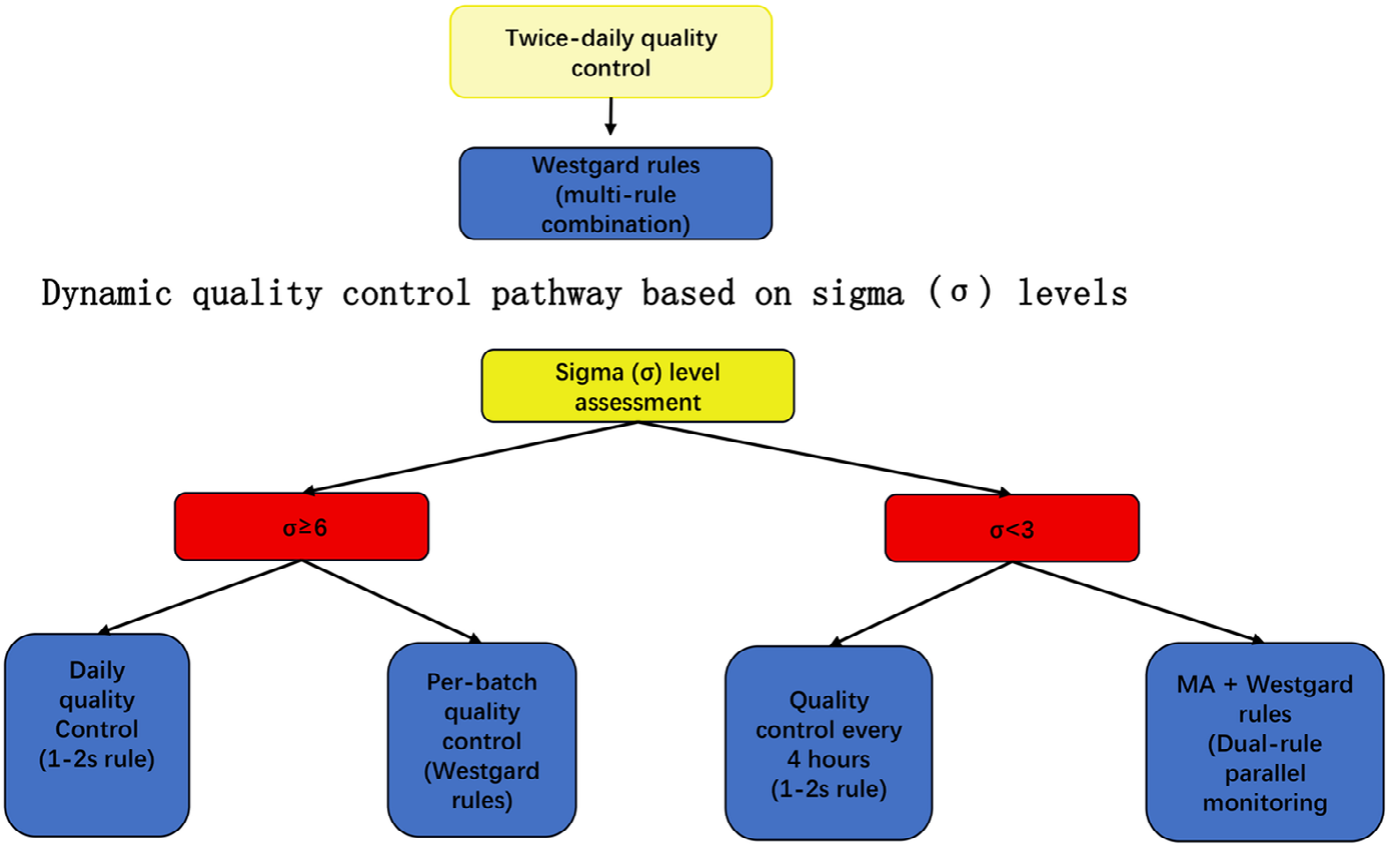
Quality control logic flowchart.

A detailed summary of *σ* values for each parameter across centers is provided in Table 1, and the corresponding dynamic QC pathway developed based on these results is illustrated in Figure 2.

**TABLE 1:** Summary of sigma (*σ*) metrics for hematology parameters across six centers.

Parameter	Hospital A	Hospital B	Hospital C	Hospital D	Hospital E	Hospital F	Mean	Sigma grade
Hb	6.72	6.91	6.55	6.33	7.05	6.68	6.71	High (≥ 6)
WBC	6.41	6.79	6.58	6.15	6.94	5.87	6.46	High (≥ 6)
RBC	5.12	4.63	5.47	4.84	5.01	5.18	5.04	Moderate (3–6)
HCT	4.85	5.32	4.12	4.47	5.06	4.53	4.72	Moderate (3–6)
PLT	3.42	3.19	2.96	2.88	3.22	3.01	3.11	Moderate

### 3.2 Application of the Dynamic QC Strategy

**FIGURE 3:**
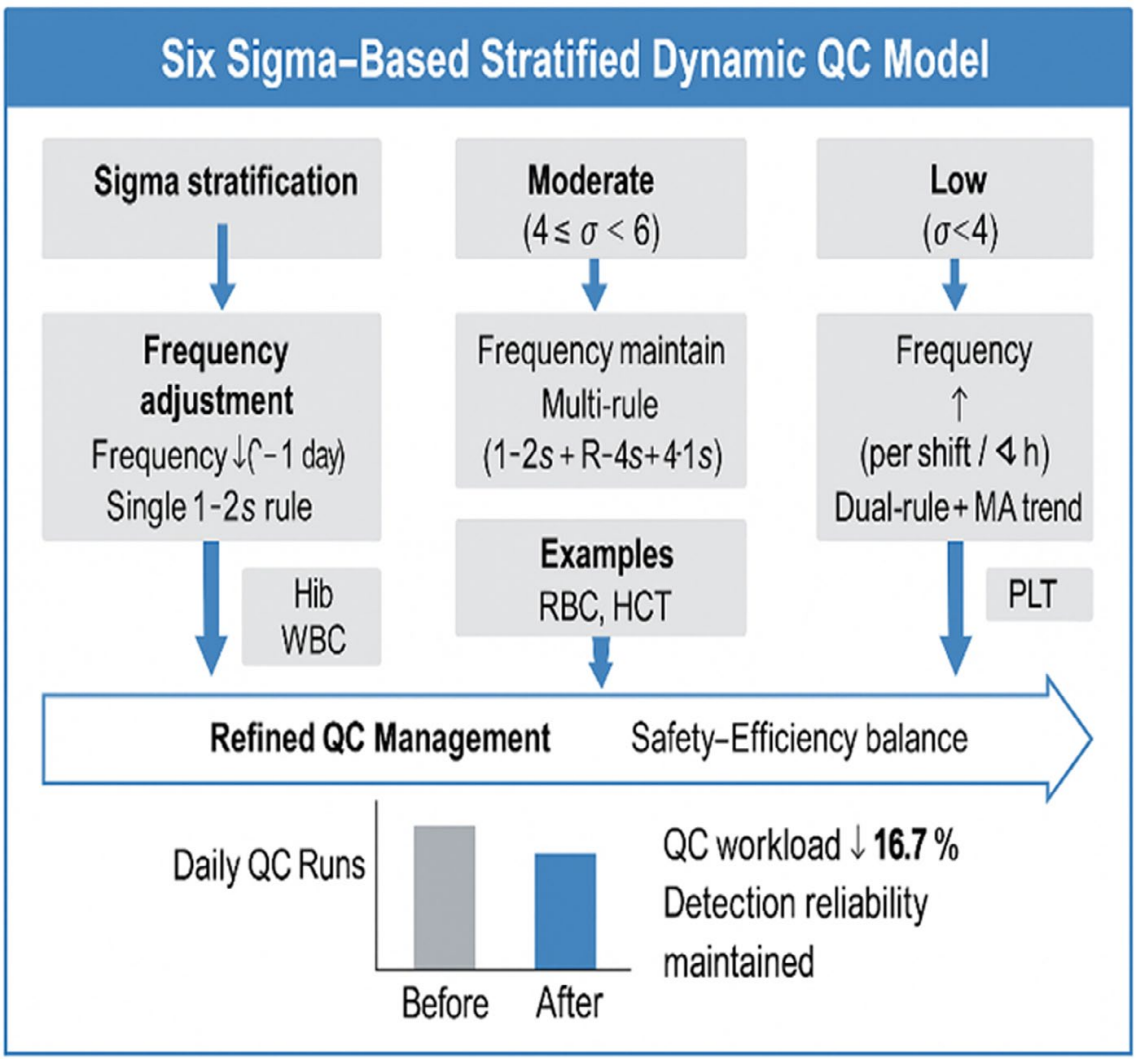
Implementation of the Six Sigma‐based stratified dynamic quality control (QC) strategy for hematology parameters on Mindray BC‐7500 series analyzers.

The implementation of the stratified dynamic QC strategy is shown in Figure 3.

Hematological parameters were grouped into three sigma‐based tiers, and corresponding adjustments were made to QC frequency and rule application.

This tiered model improved the efficiency of routine QC management while maintaining diagnostic reliability, resulting in a 16.7% decrease in QC material consumption across six participating centers.

### 3.3 Comparison of QC Effectiveness Before and After Intervention

**FIGURE 4:**
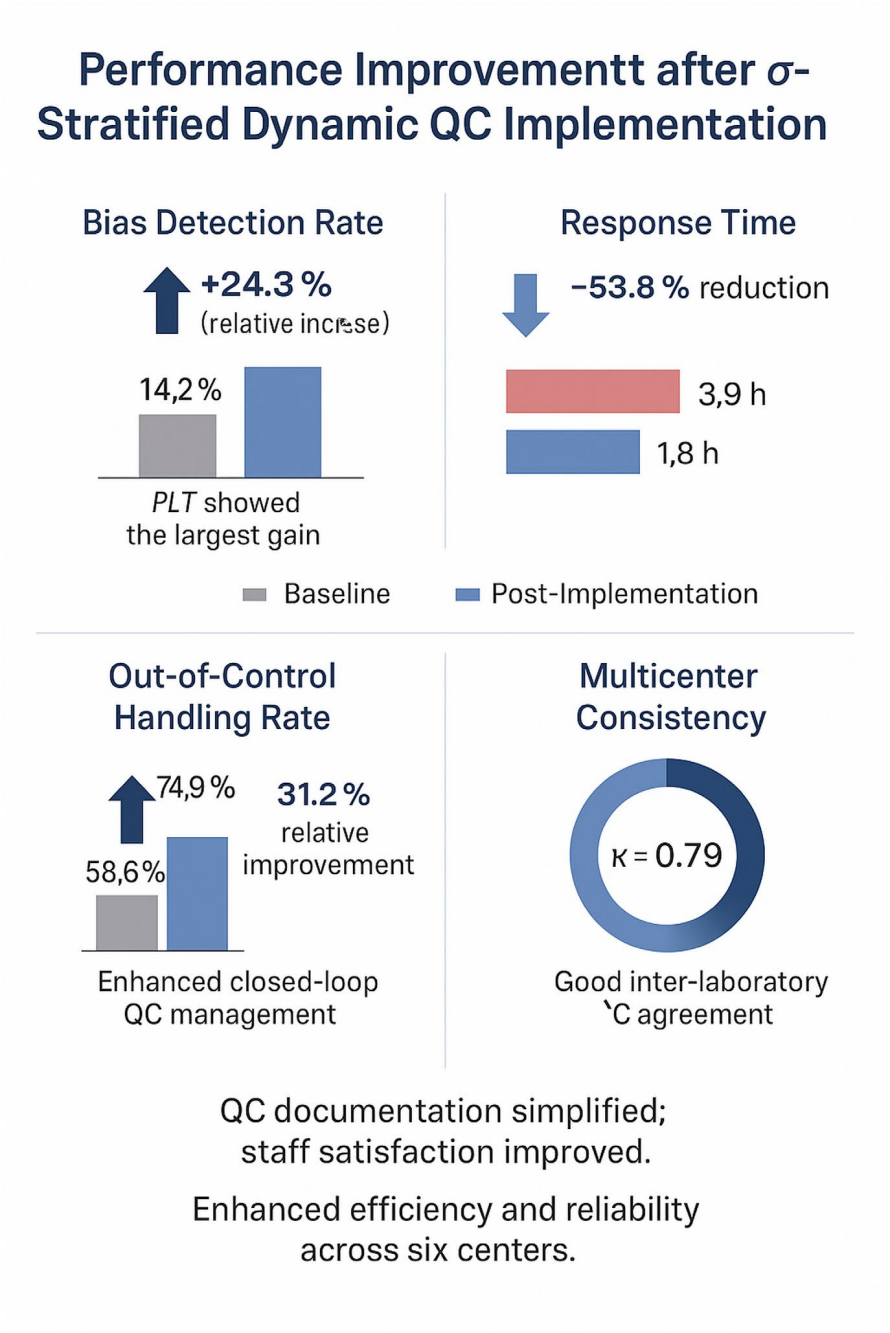
Performance improvement following implementation of the *σ*‐stratified dynamic quality control (QC) strategy across six participating centers.

Figure 4 summarizes the overall performance improvements after implementation of the *σ*‐stratified dynamic QC strategy.

Across six participating centers, measurable gains were observed in all major indicators. The bias detection rate increased from 14.2% to 17.7% (+24.3%), indicating greater sensitivity to systematic deviation, particularly in platelet (PLT) analysis. The mean response time from QC alert to corrective action decreased from 3.9 h to 1.8 h (−53.8%), demonstrating faster issue resolution. The proportion of out‐of‐control events properly handled rose from 58.6% to 76.9% (+31.2%), reflecting improved closed‐loop QC management. Inter‐laboratory agreement reached κ = 0.79, supporting the reproducibility of the strategy across heterogeneous laboratory settings. In addition, most centers reported simplified QC documentation and higher staff satisfaction, suggesting better operational efficiency and resource utilization.

**FIGURE 5:**
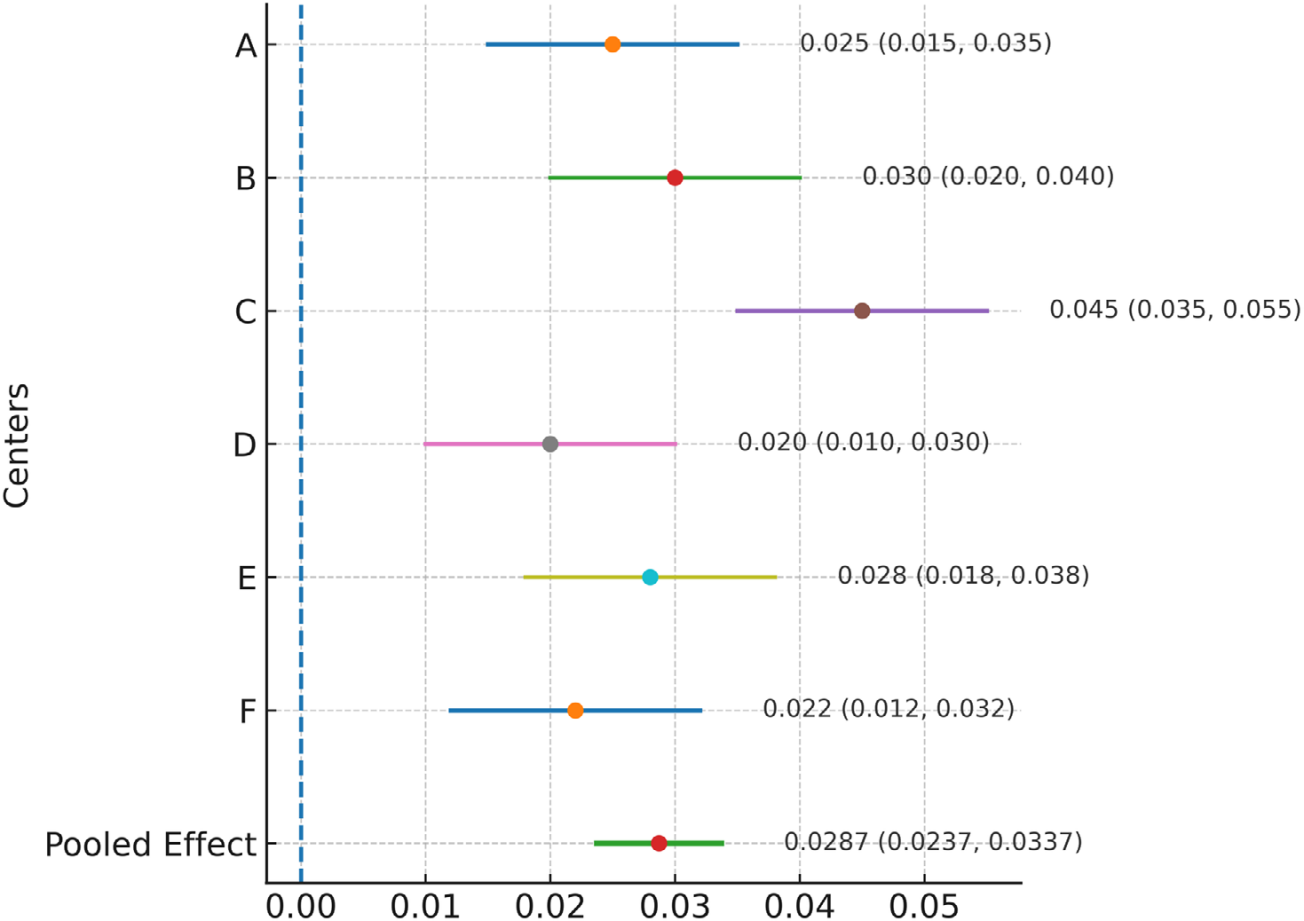
Forest plot of improvement in bias detection rate across six centers and pooled effect.

To further illustrate the multicenter effect, comparative analyses were visualized in a forest plot. The results showed a consistent direction of improvement across all six centers, with the main difference being the magnitude of improvement. The pooled effect was statistically significant (*p* < 0.01) with a narrow 95% CI, indicating that the strategy was both robust and broadly applicable. These findings were concordant with the Kappa statistic (κ = 0.79), which demonstrated moderate‐to‐high inter‐center agreement (Figure 5).

### 3.4 Performance of MA‐ and LSTM‐Assisted Early Warning

Using platelet (PLT) as a representative parameter, the LSTM model achieved a prediction accuracy of 89.7% for the mean deviation trend of the next ten samples (Table 2). The mean absolute error (MAE = 3.74 × 10^9^/L) was substantially lower than the total allowable error (TEa, ±10 × 10^9^/L), confirming stable predictive precision.

**TABLE 2:** Early‐warning performance of the MA algorithm for PLT (*n* = 69 alerting events).

Warning type	Number of events	Westgard verification result	Corrective action
True out‐of‐control (confirmed)	57	Rule violation	Instrument recalibration/reagent replacement
False alarm	12	Rule passed	Continued testing; MA parameter optimization

*Note:* Data were collected from Centers A, C, and D during the period from October 2023 to March 2024.

Receiver operating characteristic (ROC) analysis demonstrated good discriminative performance, with an AUC of 0.873 (95% CI: 0.812–0.927) when predictive deviation exceeded 50% of Tea (Table 3).

**TABLE 3:** Discriminatory performance of the MA algorithm in PLT early warning.

Metric	Value
Sensitivity	82.6% (57/69)
Specificity	92.3% (96/104)
Positive predictive value (PPV)	82.6%
Negative predictive value (NPV)	92.3%
AUC (95% CI)	0.91 (0.85–0.96)

*Note:* Data were obtained from MA‐based screening and Westgard verification of PLT results across Centers A, C, and D (*n* = 173) during the period from October 2023 to March 2024.

The average lead time between the LSTM‐predicted deviation and the first MA‐triggered anomaly was 3.2 h, allowing QC personnel to respond proactively before analytical deviations propagated.

**FIGURE 6:**
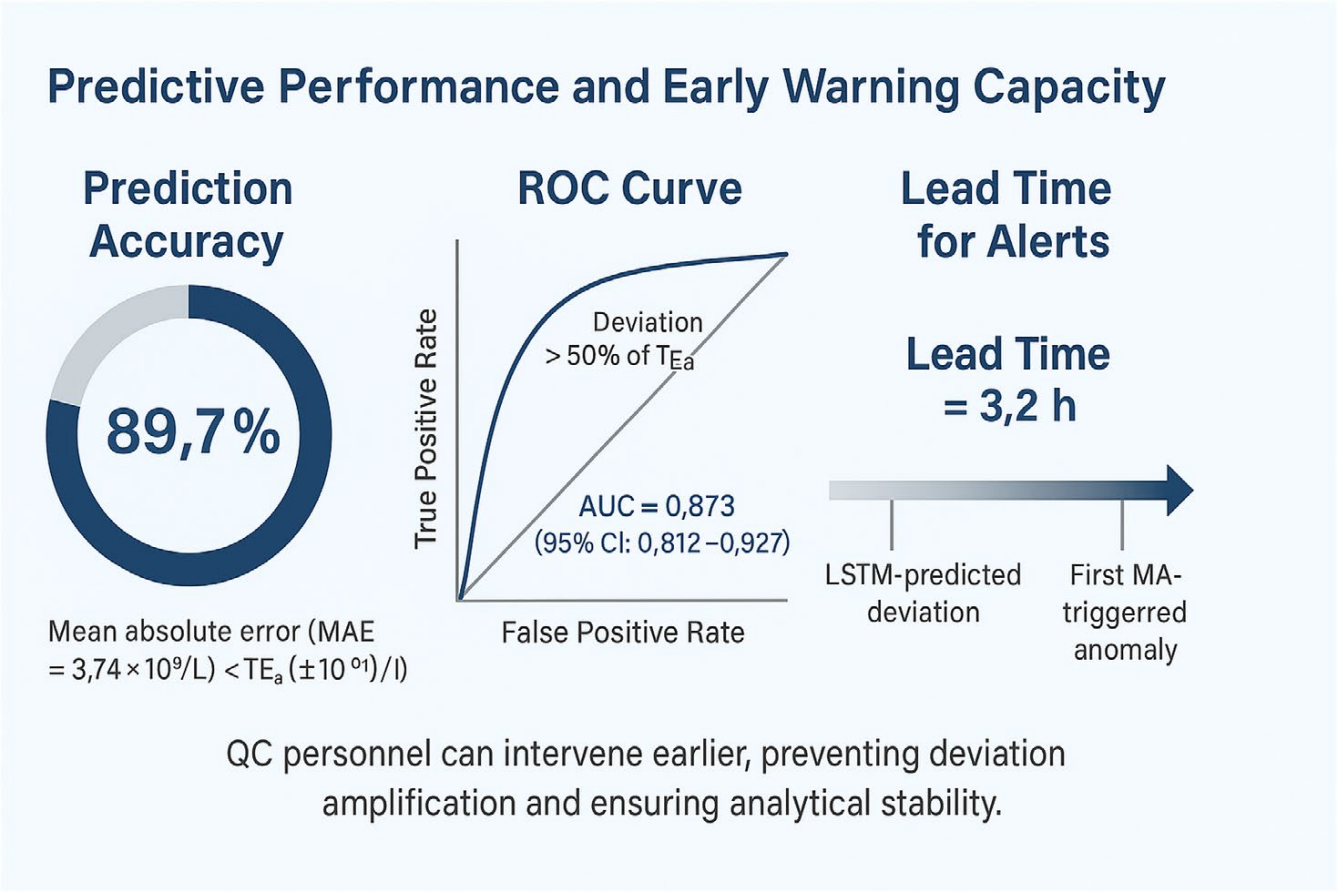
Predictive performance and early‐warning capacity of the LSTM–MA combined quality control (QC) model for platelet (PLT) analysis.

These findings indicate that integration of LSTM prediction with MA monitoring substantially enhances the anticipatory capacity of the QC system and supports early bias correction in hematology testing (Figure 6).

**FIGURE 7:**
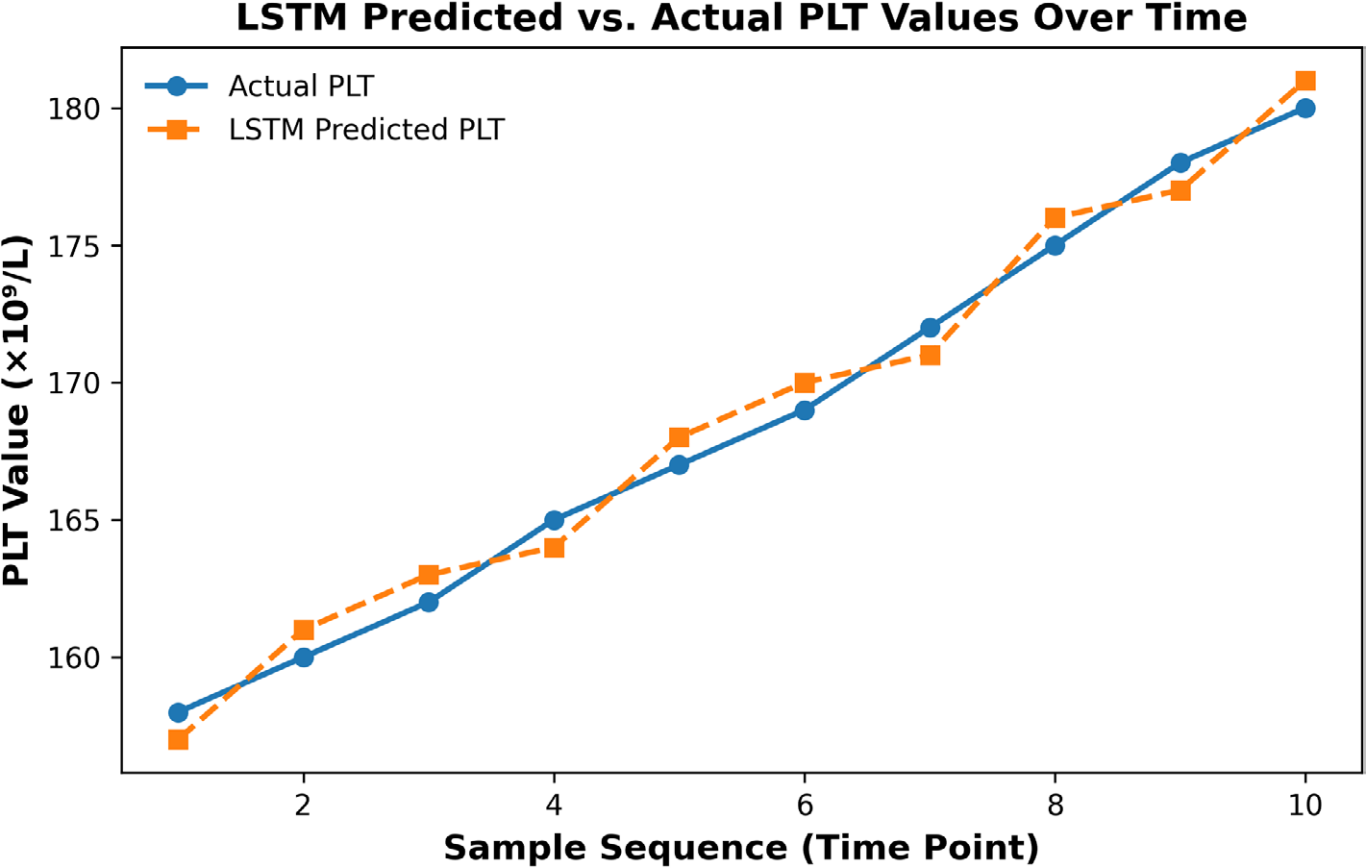
Comparison of LSTM‐predicted and actual PLT values. The figure shows the predicted trend of platelet (PLT) values over 10 consecutive samples using an LSTM model (dashed line), compared with actual measurements (solid line). The close alignment between predicted and observed values demonstrates the model's potential in forecasting systemic shifts for early warning in dynamic quality control.

The comparison between LSTM‐predicted curves and actual deviation trajectories is illustrated in Figure 7.

### 3.5 Comparison of MA and Westgard Collaborative Early‐Warning Mechanisms

To further evaluate the independent and combined effectiveness of the moving average (MA) algorithm and Westgard rules in QC early warning, a retrospective subgroup analysis was performed on PLT QC alerting events collected from the three centers implementing MA monitoring (A, C, and D) between October 2023 and March 2024.

Based on the triggering mechanism, early‐warning events were categorized into two groups:

Westgard‐only trigger: alerts directly generated by conventional rules (e.g., 1–2 s, R–4 s).

MA + Westgard combined trigger: alerts initially generated by MA monitoring and subsequently confirmed by Westgard rules.

The analysis demonstrated that the combined trigger mechanism outperformed Westgard alone in both sensitivity and positive predictive value (PPV):

Sensitivity: 89.5% for the combined mechanism versus 68.4% for Westgard alone.

PPV: 89.5% for the combined mechanism versus 75.0% for Westgard alone.

In addition, the average lead time of MA‐triggered alerts was approximately 0.7 h earlier than Westgard alone, underscoring its capacity for proactive detection in high‐throughput hematology testing. This advantage effectively shortened the lag in corrective actions and reduced the risk of error propagation due to undetected analytical shifts.

Taken together, the synergistic logic of “real‐time trend recognition via MA + specificity verification via Westgard rules” demonstrated substantial practical value in QC early‐warning systems. This combined model not only improved the accuracy of early shift detection but also reduced false alarms, providing a feasible technical pathway for laboratories to establish proactive and stratified risk‐intervention strategies under resource‐limited conditions.

The detailed comparative results are presented in Table 4.

**TABLE 4:** Comparison of early‐warning performance: MA + Westgard combined vs. Westgard‐only rules.

Metric	Westgard only	MA + Westgard combined
Number of alerting events	52	38
True out‐of‐control events	39	34
False positives	13	4
Sensitivity (%)	68.4%	89.5%
Specificity (%)	91.2%	95.8%
Positive predictive value (PPV, %)	75.0%	89.5%
Negative predictive value (NPV, %)	88.5%	95.8%

*Note:* True out‐of‐control events were confirmed by Westgard rule verification and manual corrective action records. Specificity was calculated based on events without alerts that were subsequently verified as normal.

### 3.6 Corrective Effect of Patient Data on *σ* Values

In the supplementary validation using patient samples, it was observed that the CV of patient‐derived PLT results (CV_patient = 3.5%) was significantly higher than that of internal QC materials (CV_IQC = 2.8%, *p* = 0.003, paired *t*‐test). This discrepancy resulted in a decline of the PLT *σ* value from 3.19 to 2.55, representing a relative reduction of 20.1%. For the other hematological parameters, no significant differences were observed between CV_patient and CV_IQC (*p* > 0.05), suggesting that the analytical stability of PLT is more vulnerable to biological variability (Table 5).

**TABLE 5:** Comparison of CVs between QC materials and patient samples and their corrective impact on *σ* values.

Parameter	CV<sub>IQC</sub > (%)	CV< sub>patient</sub> (%)	Correction factor (F)	Original *σ*	Corrected *σ*
PLT	2.8	3.5	1.25	3.19	2.55
HCT	1.5	1.6	1.07	4.65	4.35
Hb	0.9	0.9	1.00	6.71	6.71

*Note:* CV<sub>IQC</sub> = coefficient of variation from internal QC materials; CV<sub>patient</sub> = coefficient of variation from patient samples. Corrected *σ* values were derived by applying the correction factor (F = CV<sub>patient</sub >/CV<sub>IQC</sub>) to the original *σ*.

Based on the corrected results, adjustments were made to the stratified QC pathway:

PLT: The corrected *σ* decreased from 3.19 to 2.55, crossing the critical threshold of *σ* = 3 defined in the dynamic QC strategy. Consequently, PLT was reclassified from a *moderate‐performance parameter* to a *low‐performance parameter*, requiring a more stringent QC protocol, that is, testing every 4 h in combination with Westgard multirules and MA‐based real‐time monitoring.

Other parameters (e.g., Hb, HCT): Minor changes in corrected *σ* values did not affect their QC classification or strategy.

These findings highlight that patient‐derived CV correction is not only a supplementary index for evaluating analytical performance but also a determinant factor for QC stratification and pathway optimization. For parameters such as PLT, which are prone to greater biological variability, incorporating patient data in *σ* value correction provides critical guidance for intervention and significantly improves the scientific validity and clinical applicability of stratified QC strategies.

### 3.7 Comparative Performance of MA, Westgard, and Their Combined Application

To systematically evaluate the independent and synergistic effects of the moving average (MA) algorithm and Westgard rules in QC early warning, a subgroup analysis was conducted on PLT QC alerting events recorded between October 2023 and March 2024 in the three centers that had implemented MA monitoring (A, C, and D).

Based on the triggering mechanism, early‐warning events were categorized into three groups:

Westgard‐only group (WG): alerts directly generated by conventional Westgard rules (e.g., 1–2 s, R–4 s).

MA‐only group (MA): alerts triggered by abnormal patient‐data trends identified by the MA model, without simultaneous Westgard confirmation.

Combined group (MA + WG): alerts initially generated by MA monitoring and subsequently confirmed by Westgard rules.

Key performance metrics—including sensitivity, false‐positive rate (1 − specificity), positive predictive value (PPV), and negative predictive value (NPV)—were compared across the three groups to assess their discriminative capabilities under practical QC conditions (Table 6).

**TABLE 6:** Discriminatory performance of MA, Westgard, and combined early‐warning mechanisms for PLT QC.

Trigger mechanism	No. of alerts	True out‐of‐control	False alarms	Sensitivity (%)	Specificity (%)	PPV (%)	NPV (%)
Westgard only	52	39	13	68.4	91.2	75.0	88.5
MA only	69	57	12	82.6	92.3	82.6	92.3
MA + Westgard	38	34	4	89.5	95.8	89.5	95.8

*Note:* True out‐of‐control events were confirmed by Westgard rule failure and subsequent corrective actions. Specificity was calculated based on events without alerts that were verified as normal.

The results demonstrated the following:

Sensitivity: The MA‐only group achieved a sensitivity of 82.6%, significantly higher than the 68.4% observed with Westgard rules alone, indicating superior ability for early trend recognition.

Combined application: Sensitivity and specificity were simultaneously elevated to 89.5% and 95.8%, respectively, with PPV and NPV also reaching 89.5% and 95.8%, representing the best overall performance among the three groups.

False‐positive control: The combined mechanism provided a dual‐layer verification of “prospective trend detection + rule‐based confirmation,” which not only enhanced predictive accuracy but also effectively reduced false alarms, thereby improving both reliability and resource efficiency.

Taken together, the synergistic application of MA and Westgard rules establishes a more robust QC pathway characterized by early screening coupled with specific verification. This dynamic QC strategy is particularly suitable for low‐*σ* parameters such as PLT and offers laboratories a forward‐looking, high‐efficiency solution for risk‐based quality management.

## 4 Discussion

Using the Six Sigma quality management model, this study systematically assessed the performance of five hematological parameters (Hb, WBC, RBC, HCT, and PLT) across multiple centers and established a dynamic QC strategy. Considerable differences in *σ* values were observed: Hb and WBC showed excellent stability (*σ* ≥ 6), reaching “world‐class” standards, while PLT consistently demonstrated poorer performance, with only a minority of centers achieving *σ* ≥ 3.5. These findings align with previous reports indicating that hematology analyzers are influenced by methodology, instrument platform, and QC material variability [5, 6, 11, 14].

The relatively low *σ* values for PLT are attributable to its methodological characteristics. PLT counting depends on cell‐size distribution and fluorescent staining, which are prone to interference from microparticles, fragments, and residual erythrocytes, leading to increased bias and variability. Moreover, differences among analytical platforms in threshold settings, amplification algorithms, and dye specificity may further contribute to systematic bias. Variability in reagent lots, calibration procedures, and maintenance practices also played a role. For instance, laser‐scattering platforms use different size thresholds, potentially underestimating platelet counts and affecting bias estimation [26, 27]. Thus, it is advisable to standardize bias estimation by adopting EQA medians or third‐party targets, with further calibration using Ricos database values [23, 24].

The Six Sigma model, which incorporates TEa, bias, and CV into a single *σ* value, has been endorsed by ISO and CLSI [8, 12, 13, 15]. Our results confirm that stratifying parameters by *σ* facilitates differentiated QC frequency and rule selection, supporting the transition from uniform to more refined quality management.

This study is the first to integrate the moving average (MA) algorithm with a long short‐term memory (LSTM) predictive model into hematology QC. MA enables continuous monitoring of patient results, compensating for the limited frequency and coverage of traditional QC materials. When deviations exceed ±1.5 SD, MA issues an alert, while Westgard rules provide confirmatory validation. Together, these form a collaborative mechanism of “real‐time detection plus targeted verification,” particularly valuable for low‐*σ* parameters such as PLT.

In practice, the use of MA reduced the average detection time for systematic PLT shifts to 0.8 h, with 82.6% of alerts subsequently confirmed by Westgard rules. This indicates that the combined approach enhances both sensitivity and response speed.

However, potential false positives remain an important consideration. These may result from differences between training data and real‐world distributions, noise amplification in small MA windows, or inter‐laboratory variability in platforms, reagent lots, and patient populations. Accordingly, laboratories implementing MA and LSTM should retrain models and optimize parameters using local datasets to improve generalizability and reduce false alarms.

The dynamic QC strategy provided clear efficiency gains. The mean number of QC operations decreased from 60 to 50 per day, while monthly QC material consumption fell by 360 runs, reducing reagent costs by approximately 720 CNY. Improved responsiveness further saved 6–8 h of staff time per month, equivalent to about 550 CNY in labor costs. In total, monthly savings amounted to ~1270 CNY.

Simplification of QC documentation also improved staff satisfaction, demonstrating that the strategy delivers both safety and economic benefit.

Several limitations should be acknowledged. First, *σ* values in this study were based on QC material CV and bias, which may not fully reflect biological variation in patient samples and are susceptible to matrix effects [5, 14]. Second, bias estimation relied on platform comparison, potentially introducing systematic error; standardized use of EQA medians or third‐party targets with Ricos‐based corrections is recommended [23, 24]. Beyond these methodological considerations, achieving comprehensive standardization in hematology testing also requires the incorporation of metrological traceability.

While the present study primarily evaluated QC performance based on commercial control materials, patient‐derived data, and EQA programs, further harmonization relies on establishing traceability to higher order reference measurement procedures and certified reference materials.

Embedding traceability‐based calibration chains within the *σ*‐stratified dynamic QC framework would allow bias and total error to be expressed in traceable units, thereby linking internal QC indicators to internationally recognized reference systems.

This approach aligns with the ICSH‐endorsed reference methods for red and white blood cell counting, which have been officially registered in the JCTLM Reference Measurement Procedure (RMP) Database (News ID 27; JSLH entry).

Integration of these traceability pathways would enhance result comparability and long‐term analytical reliability across laboratories, supporting the global harmonization of hematology quality control practices. Third, the observation period was only 6 months, limiting assessment of seasonal variation, staff turnover, and long‐term instrument stability. Future studies should extend the monitoring period.

Although an LSTM model was included, MA parameter selection remained partly subjective. Adaptive approaches, such as reinforcement learning or Bayesian optimization, may improve robustness and applicability [19, 20]. Finally, QC response after alerts still depended on manual intervention; integrating LIS‐based closed‐loop management could enhance automation, efficiency, and traceability.

The proposed multitier strategy—*σ* stratification combined with MA monitoring and LSTM prediction—closely aligns with international quality management frameworks. The U.S. CLIA IQCP emphasizes risk‐based QC tailored to method, staff, and instrument characteristics [22], while CLSI EP23‐A promotes individualized, risk‐oriented QC [13]. By quantifying performance with *σ* values and embedding AI‐driven real‐time monitoring, our approach reflects the global trend of “risk stratification matched with targeted strategy.”

Compared with traditional “one‐size‐fits‐all” QC, this strategy ensures safety while reducing redundancy, making it scientifically robust and economically efficient. It is particularly suitable for primary laboratories and provides practical insights for regional QC integration.

Multicenter analysis confirmed the adaptability of this dynamic QC model. A Kappa coefficient of 0.79 indicated strong consistency across centers, supporting its generalizability. Future work should leverage regional QC platforms to develop a *σ* value–frequency–intervention management matrix, enabling standardized, precision‐driven laboratory management.

With greater integration of AI, big data, and LIS, intelligent rule selection and real‐time optimization of QC monitoring will be the next step. For resource‐limited settings, simplified schemes (e.g., fixed‐window MA with a single Westgard rule), supported by regionally standardized LSTM parameters, could enable broader implementation.

At the same time, the ongoing shift from QC material‐based paradigms to patient‐based QC (PBQC) underscores the importance of patient data as a complement or alternative to traditional controls. In high‐throughput testing such as hematology, patient‐based monitoring is more sensitive to systematic shifts and instrument drift, overcoming the limitations of QC materials. By incorporating patient‐derived CV correction, MA trend modeling, and LSTM forecasting, this study demonstrates the transition of patient data from a validation tool to a sentinel monitoring mechanism, improving the relevance and responsiveness of QC. Future research should explore large‐scale PBQC models with integrated feedback, providing more precise, safe, and cost‐effective solutions for laboratory quality management worldwide.

## Author Contributions

Ming Hu: Conceptualization, Methodology, Supervision, Writing – Review and Editing. Bo Liu and Zhaodong Sun: Investigation, Data Curation, Formal Analysis, Writing – Original Draft. Kaiyong Chen: Resources, Project Administration. Min Liu: Data Curation, Statistical Analysis. Jibao Qin and Dengli Feng: Resources, Investigation. Fumeng Yang: Project Administration, Supervision. Jiaping Wang and Huiyi Wu: Data Curation, Software, Validation. All authors have read and approved the final manuscript.

## Funding

The authors have nothing to report.

## Conflicts of Interest

The authors declare no conflicts of interest.

## Data Availability

The data that support the findings of this study are available from the corresponding author upon reasonable request.
